# 

**Published:** 1911-08

**Authors:** 


### LOVE.

That attribute of our nature we call "love," and which, with civilized people, is the tie that binds the family and society, is the highest, noblest and most beautiful that actuates man. It is the mainspring of human ambition and endeavor. Upon it are founded marriage, home, the family and social order. It prompts to the acquisition of fame and fortune, the founding of the home, and the rearing of a family, the upbuilding of society and civilization. Its physical and psychic basis is that instinct which God has implanted in every animal creature, from the moneron to man, to insure a perpetuation of the species. Around this powerful and compelling impulse sentiment has woven the most beautiful and exalting emotions of the heart,—sympathy, friendship, affection, pity, mercy and charity. It has been the theme of the poet in all ages. Stripped of these sentiments it stands revealed in the hideous form of lust,—animal desire, brute passion, as primitive man and his progenitors knew it,—like a rugged rock, around which, to conceal its monstrous form, nature has woven the tendrils and foliage and flowers of a beautiful clinging creeper. Divested of these refined and refining features, and unrestrained by the many inhibitions which society, law, religion and ethics have placed upon it, it is a power for evil,—an unchained demon, a cyclone, an irresistible force, which, in the history of the world and the evolution of man, has wrought untold woe and misery.

FERDINAND EUGENE DANIEL.

Austin, Texas.

